# Adsorption of Omeprazole on Biobased Adsorbents Doped with Si/Mg: Kinetic, Equilibrium, and Thermodynamic Studies

**DOI:** 10.3390/molecules28124591

**Published:** 2023-06-06

**Authors:** Roberta A. Teixeira, Pascal S. Thue, Éder C. Lima, Alejandro Grimm, Mu. Naushad, Guilherme L. Dotto, Glaydson S. dos Reis

**Affiliations:** 1 Graduate Program in Water Resources and Environmental Sanitation, Hydraulic Research Institute (IPH), Federal University of Rio Grande do Sul (UFRGS), Porto Alegre 91501-970, RS, Brazil; roberta.arleu@gmail.com; 2 Environmental Science Graduate Program, Engineering Center, Federal University of 8 Pelotas (UFPel), 989 Benjamin Constant St., Pelotas 96010-020, RS, Brazil; pascalsilasthue@gmail.com; 3 Institute of Chemistry, Federal University of Rio Grande do Sul–UFRGS, Av. Bento Gonçalves 9500, P.O. Box 15003, Porto Alegre 91501-970, RS, Brazil; profederlima@gmail.com; 4 Department of Forest Biomaterials and Technology, Biomass Technology Centre, Swedish University of Agricultural Sciences, SE-901 83 Umeå, Sweden; alejandro.grimm@slu.se; 5 Department of Chemistry, College of Science, King Saud University, P.O. Box 2455, Riyadh 11451, Saudi Arabia; shad123@gmail.com; 6 Research Group on Adsorptive and Catalytic Process Engineering (ENGEPAC), Federal University of Santa Maria, Av. Roraima, 1000-7, Santa Maria 97105–900, RS, Brazil; guilherme_dotto@yahoo.com.br

**Keywords:** wood waste, magnesium carbon dopant, silica carbon dopant, adsorption, omeprazole

## Abstract

This paper proposes an easy and sustainable method to prepare high-sorption capacity biobased adsorbents from wood waste. A biomass wood waste (spruce bark) was employed to fabricate a composite doped with Si and Mg and applied to adsorb an emerging contaminant (Omeprezole) from aqueous solutions, as well as synthetic effluents loaded with several emerging contaminants. The effects of Si and Mg doping on the biobased material’s physicochemical properties and adsorptive performance were evaluated. Si and Mg did not influence the specific surface area values but impacted the presence of the higher number of mesopores. The kinetic and equilibrium data presented the best fitness by the Avrami Fractional order (AFO) and Liu isotherm models, respectively. The values of Q_max_ ranged from 72.70 to 110.2 mg g^−1^ (BP) and from 107.6 to 249.0 mg g^−1^ (BTM). The kinetic was faster for Si/Mg-doped carbon adsorbent, possibly due to different chemical features provoked by the doping process. The thermodynamic data showed that the adsorption of OME on biobased adsorbents was spontaneous and favorable at four studied temperatures (283, 293, 298, 303, 308, 313, and 318 K), with the magnitude of the adsorption correspondent to a physical adsorption process (ΔH° < 2 kJ mol^−1^). The adsorbents were applied to treat synthetic hospital effluents and exhibited a high percentage of removal (up to 62%). The results of this work show that the composite between spruce bark biomass and Si/Mg was an efficient adsorbent for OME removal. Therefore, this study can help open new strategies for developing sustainable and effective adsorbents to tackle water pollution.

## 1. Introduction

Nowadays, the pollution of water bodies is a tremendous issue for the environment, animals, and human beings, and due to the intense human and industrial activities, this issue has only grown [1,2]. Among many different pollutants in natural waters, a new class, so-called emerging contaminants (ECs), has gathered enormous concerns from environmental researchers due to their deleterious effects on living beings, even at very small concentrations [3,4]. ECs are different compounds, such as pharmaceuticals and personal care products, plasticizers, food additives, wood preservatives, laundry detergents, surfactants, disinfectants, pesticides, hormones, etc. [3]. ECs are unregulated or not completely regulated compounds, even in the most developed countries; therefore, they must be properly removed from wastewater before being discharged into the environment. However, ECs cannot be easily removed from wastewater under traditional wastewater treatment techniques [3,4].

There are several methods employed to treat effluents loaded with ECs, including advanced oxidation procedures [5], biological treatment [6], photocatalysis [7], filtration [8], and adsorption [9,10,11]. However, most of these treatment methods are not techno-economically feasible for field implementation. Furthermore, these developed methods have some problems due to the complex procedures, maintenance, high investment cost, toxic byproduct generation, etc. In addition, they can generate toxic sludges, which need further costs for sludge management and disposal [5,6,7]. Therefore, a more suitable treatment process for removing ECs is required, and adsorption appears as one of the most suitable treatment methods due to its high-efficiency removal, low initial implementation and operational costs, easy operation, and controllable residue generation.

In order to achieve an effective adsorption process, selecting and designing a suitable and efficient adsorbent must be met [12,13,14]. Among the most popular adsorbents, activated carbon (AC) is widely popular and effectively employed [12,13,14]. Activated carbons are well-known for their excellent adsorption characteristics due to their highly developed pore structure, elevated surface area, and essential surface activity [12,13]. However, ACs possess the disadvantages of relatively complex synthesis processes—mainly during the activation step—that usually employ high amounts of chemicals such as zinc chloride and potassium hydroxide (usually a ratio of 1:1 of chemical/biomass) [15,16,17,18]. Moreover, a washing step with hydrochloric acid is needed to remove the remaining chemical activator after the pyrolysis process [19,20,21,22]. These steps are directly correlated with the high costs of activated carbon preparation.

Each biobased carbon adsorbent property deeply depends on the pyrolysis conditions and chemical/dopant used, as theses impact its adsorptive performances due to enhancing its active surface sites [16,17]. For instance, doping the carbon matrix with Si and Mg increases adsorptive active sites [18]. This work prepared biobased adsorbent materials using biomass wood waste (spruce bark). As far as we know, for the first time, we report the preparation of new composite biobased materials doped with Si and Mg and subsequently used to adsorb an emerging contaminant (Omeprezole) from aqueous solutions. Furthermore, the effect of doping on the biochar’s final physicochemical properties was subjected to a deep investigation. This paper successfully improves sustainable strategies for developing high-performance biomass-based adsorbents to remove EC from wastewater. This research is expected to help open new strategies for developing sustainable and effective adsorbents to tackle water pollution.

## 2. Results and Discussion

### 2.1. Characterization of the Adsorbents

Textural properties such as SSA and porosity (meso- and micropore features) are very important for determining any adsorbent’s quality that will impact its adsorptive performances [22,23]. The textural characteristics of the biobased adsorbents (BP and BTM) are summarized in Table 1. It shows that the chemical dopants used did not influence the SSA value. Both samples exhibited SSA values of 205 and 202 m^2^ g^−1^ for BP and BTM, respectively. Compared with the literature, Deng et al. [24] prepared corncob biochars doped with MgCl_2_ and obtained specific surface areas ranging from 52.41 to 174.29 m^2^ g^−1^. Although there was no difference in SSA values, the doping Si/Mg did influence the mesopore contribution compared to the pristine biobased carbon. The mesopores are responsible for adsorbing molecules larger in size, and also it could improve the uptake of smaller molecules since each pore of the material can accommodate agglomerates of small molecules [12,14]. However, the results show that both adsorbents are highly microporous, which is a good characteristic that may facilitate the diffusion of smaller adsorbate molecules such as OME into the adsorbents’ pores [25].

**Figure 1:**
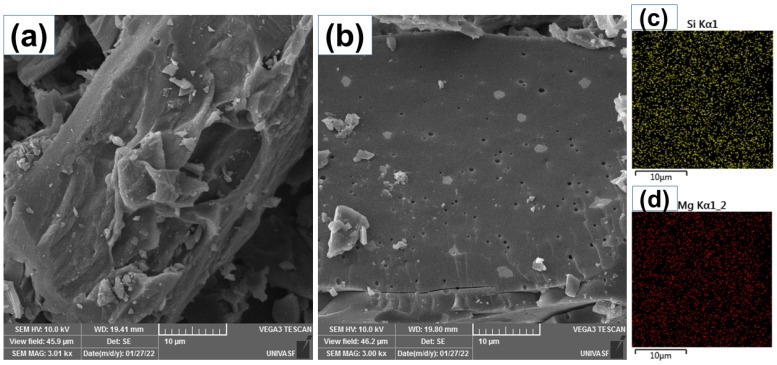
SEM images of (**a**) BP; (**b**) BTM; (**c**) Si mapping for BTM, and (**d**) Mg mapping for BTM.

**Figure 2:**
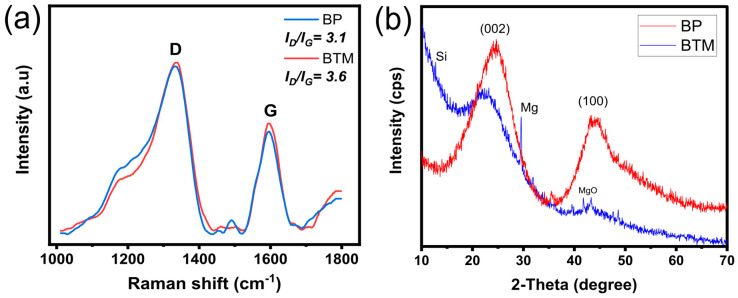
Raman spectra (**a**) and XRD patterns (**b**) of the adsorbent materials.

The surface morphological characteristics of BP and B-Si-Mg adsorbents are evaluated by SEM images (shown in Figure 1). BP exhibits a more irregular surface with no apparent porosity. In contrast, BTM showed a smoother surface with several small holes unveiling some developed porous structures related to a high surface area. On the doped biochar’s surface, it is possible to observe some white dots, which are the dopants attached (fused) to the biochar carbon matrix. It is reported that adsorbents rich in both physical (irregular morphology) and chemical (by introducing Si/Mg atoms) defects can be beneficial in adsorbing OME because these defects act as adsorptive sites that can boost the adsorption through pore filling and electrostatic interaction (further explained ahead in the mechanism of adsorption) [11,13,17]. Complementarily to SEM, EDS mapping of Si (Figure 1c) and Mg (Figure 1d) is also explored. EDS quantification of the elements indicated 18.4% and 10.7% of Si and Mg, respectively. The elements mapping of the composite surface confirmed the presence of two elements (Si and Mg), showing their uniform distribution over the carbon structure (see Figure 2c,d).

Figure 2 shows the Raman analysis for both samples; Raman spectroscopy gives valuable information on the order/disorder and degrees of biochars graphitization [26,27]. Figure 2 shows that for both samples, two characteristic peaks at 1335 cm^−1^ (D-band) and 1600 cm^−1^ (G-band). The D-band is related to C atoms of defects or disordered structures, typically dominant in amorphous carbon materials derived from biomass, while G-band represents carbon atoms with an sp^2^ electronic configuration in graphite structures [28,29,30]. In addition, from the Raman spectrum, the ratio between D and G peaks (I_D_/I_G_) is often employed to determine the degree of graphitization of the biochar.

The carbon material doped with the Si/Mg presented a slightly higher I_D_/I_G_ (3.6) compared to pure carbon sample BP (3.2) (see Figure 2a), indicating little more defects in its structure. Many reports suggest that adsorbents rich in defects, which could mean more adsorption sites, can have their adsorptive properties boosted [11]. As previously mentioned, these defects can be both physical and chemical, which are responsible for boosting the adsorbent adsorptive properties through different mechanisms (pore filling and electrostatic interactions, further explained ahead in Section 2.5). Therefore, Si/Mg dopants helped increase the adsorption sites in the BTM adsorbent.

Figure 2b shows the X-ray diffraction pattern of the adsorbent materials. Important differences are observed between both patterns (see Figure 2b). BP shows two prominent broad peaks at 2θ = 24.5° and 44°, which are associated with the (002) and (100) planes, respectively [31,32]. The (002) peak is attributed to the packed carbon layers and amorphous and aliphatic structures [31,32]. The broad peak at 44° is associated with (100) diffractions of graphitic carbons, indicating a certain degree of aromatization in the BP structure. However, for the BTM, the broad peak at (100) disappeared, indicating a higher degree of amorphization, possibly provoked by the incorporation of Si [32]. In addition, however, some small crystalline peaks can be seen related to MgO [24].

### 2.2. Kinetic of OME Adsorption on BP and BTM Adsorbents

**Figure 3:**
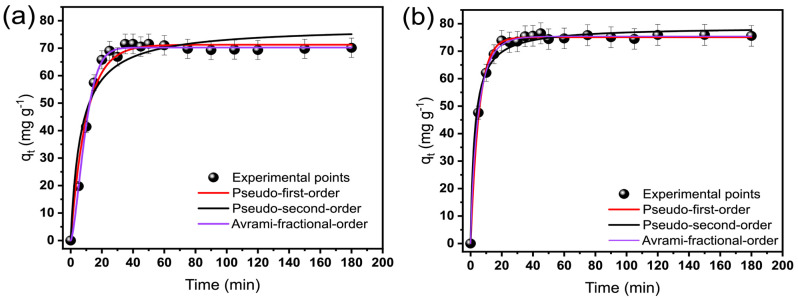
OME kinetic of adsorption curves on BP (**a**) and BTM (**b**) adsorbents. Conditions: adsorbate concentration of 300 mg L^−1^, temperature of 25 °C, adsorbent dosage of 1.5 g L^−1^, and initial solution pH 7.0.

In an adsorption process, the kinetic is a crucial step to ensure that the system reaches the saturated adsorption capacity and for understanding the mechanism(s) acting in the process regarding diffusion and mass transport in the adsorption system [29,30,31,32,33]. Figure 3 and Table 1 show the kinetic curves and parameters for OME removal onto biobased adsorbents, respectively. The same trend was observed for both adsorbents, with a rapid increase in their uptake within 25 min, which seemed to reach the equilibrium (see Figure 3). The rapid equilibrium highlights the high affinity between adsorbents and adsorbate.

The nonlinear pseudo-first-order (PFO), pseudo-second-order (PSO), and Avrami-fractional-order (AFO) models were used to analyze the fitness of the kinetic data (see Table 2 and Figure 3). It is essential to understand the adsorption rate to design the adsorbents’ application. In this study, nonlinear models were used, which offer a good fitting of the data. PFO and PSO models suppose that the interaction between adsorbate and adsorbent can be physical or chemical adsorption, following an adsorption kinetic of first or second order, respectively. However, the AFO model suggests a fractional order of the adsorption kinetic. The models’ fitness was evaluated using the adjusted R^2^_adj_, SD, and BIC values [14,25,34] (see Supplementary Materials).

Lower SD and higher R^2^_adj_ values suggest that the q experimental values are much closer to q provided by the models. Thus, it better describes the experimental values, suggesting the model’s better fitness. As seen in Table 2, AFO exhibited the highest R^2^_adj_ and lowest SD values and, therefore, better suitability to describe the kinetic adsorption of OME on both adsorbents. Moreover, the Bayesian Information Criterion (BIC) was also employed to corroborate the suitability of the kinetic model. Lima et al. (2021) [21] reported that if the difference between two BIC values (ΔBIC) of the models is ≤2.0, it means that the difference between the two models is not significant. For ΔBIC ≥ 10, it can be stated without any doubt that the model with a lower BIC value presented the best suitability. Table 2 shows that the ΔBIC values between the AFO model and PFO were 33.29 and 8.57, and the BIC differences between the AFO model and PSO were 56.71 and 33.33 for BP and BTM, respectively. This confirms that the AFO kinetic model for the OME molecules adsorption is the best-fitted. The adsorption order for the OME molecules on BP was 1.513, a value closer to 2, while n_AV_ for the adsorption of OME molecules onto BTM was 0.8409, a value close to 1.

The AFO is widely reported in the literature, and it suggests a complex process with multiple adsorption pathways, with dynamic/different mechanism (s), while adsorption occurs [35,36]. This process presents multiple kinetic orders instead of one, represented by n_AV_, which usually has a fractional value [35,36]. Thus, evaluating t_1/2_ and t_0.95_ becomes important for comparing different kinetic models (see Table 2) [19,20,25,33,34,35]. The t_0.95_ is the time to reach 95% of total saturation, and t_1/2_ is the time to reach 50%. Considering that the AFO presented the best fitness, its values for t_1/2_ and t_0.95_ were 8.32 min and 21.87 min, and 3.27 min and 18.65 min for BP and BTM adsorbents. It shows that the kinetic was faster for BTM, possibly due to different chemical features provoked by the doping with Si/Mg [24]. Short times to attain 95% saturation are obtained for carbon-based materials to uptake emerging organic molecules [12,14,20].

### 2.3. Equilibrium Isotherms Studies of OME on Biobased Adsorbents

The isotherm of adsorption is a valuable tool for better evaluating the adsorption system between the adsorbent and adsorbate [37,38,39,40,41]. There are many isotherm models, and in this work, the Langmuir, Freundlich, and Liu models were employed to study the equilibrium of adsorption between adsorbents and OME molecules [37,42,43,44]. Freundlich’s model assumes multiple layers of adsorbate covering the adsorbent. As a result, no saturation of the adsorbent is achieved, and the energy of each active site is not homogeneous, while Langmuir almost assumes the opposite. Liu is the combination of Freundlich and Langmuir models and considers the saturation of the adsorbent, a multilayer of the adsorption of adsorbate, and the activate sites of the adsorbents with different energy [39,40,44].

**Figure 4:**
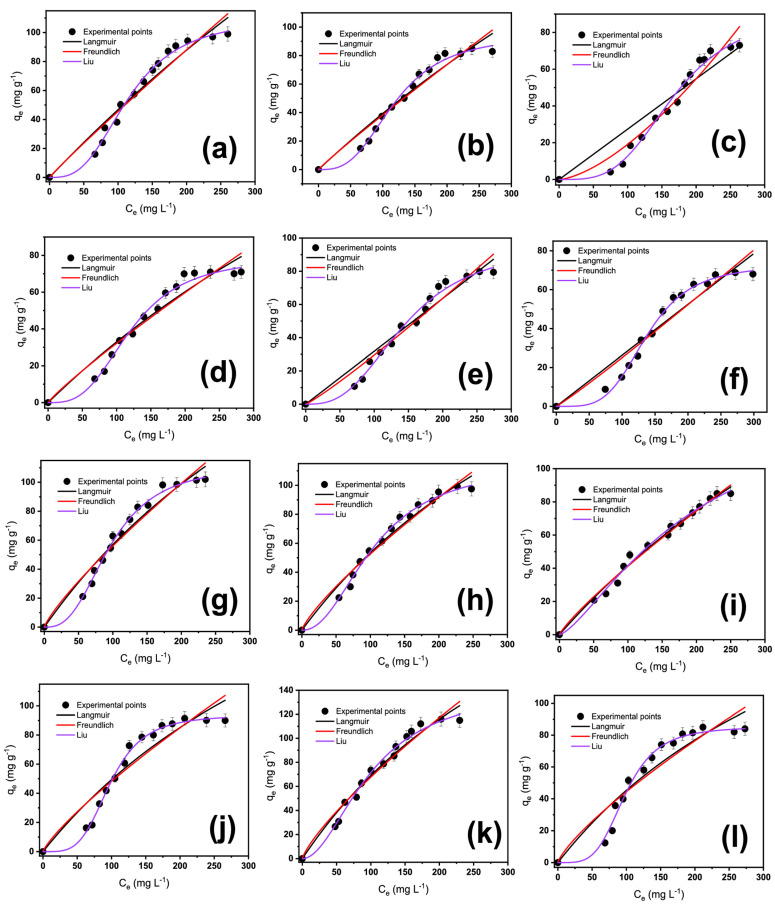
OME isotherms of adsorption curves at different temperatures for BP, (**a**) 283 K, (**b**) 293 K, (**c**) 298 K, (**d**) 303 K, (**e**) 313 K, and (**f**) 318 K; and for BTM (**g**) 283 K, (**h**) 293 K, (**i**) 298 K, (**j**) 303 K, (**k**) 313 K, and (**l**) 318 K. Conditions: Adsorbent dosage of 1.5 g L^−1^, and initial pH solution 7.0.

The isotherm curves and parameters are displayed in Figure 4 and Table 3. The fitness of the isotherm models was examined previously for the kinetics (by R^2^_adj_, SD, and BIC values). Considering the SD values and R^2^_adj_ obtained from the nonlinear isotherm model fitting, high differences were observed between the models for all temperatures. In general, Liu’s model provided the lowest SD and highest R^2^_adj_ values for both adsorbents. Moreover, taking into account the BIC values, they were lowest for Liu, which is statistically the best-chosen model [14,20,34]. The Liu model assumes that the adsorbent’s adsorption active sites cannot have the same energy. Therefore, the adsorbents may present active sites preferred by the OME molecules for occupation. However, at the studied condition, the saturation of the active sites should occur, unlike in the Freundlich isotherm model. Except for the isotherm at 298 K, Table 3 shows a fitting for the Liu isotherm model. This may indicate that the surface of the biobased adsorbents is likely heterogeneous, with different energy adsorption sites.

### 2.4. Thermodynamic Studies of OME on BP and BTM

The thermodynamics was examined using the van’t Hoff approach [22], whose equilibrium constant was accessed from the best equilibrium constant obtained in the isotherms from 283 to 318 K. Table 4 shows the thermodynamic adsorption parameters for OME on biobased adsorbents. The thermodynamic data show that the adsorption process was spontaneous and favorable at the six studied temperatures (283, 293, 298, 303, 308, 313, and 318 K), with negative values of ΔG° [20,33,34,45]. Furthermore, the adsorption process was endothermic for BTM adsorbent (ΔH° < 0) and exothermic for BP (ΔH° > 0) [20,33,45]. The ΔH° values refer to the magnitude of the adsorption [20,33,35,45], which corresponds to a physical adsorption process since its values are <40 kJ mol^−1^ [35,45]; however, other mechanisms could be involved in the adsorption of OME on BP and BTM, which is further studied in the next section. Finally, for both adsorbents, the changes in the entropy (ΔS°) were positive, which indicates an increase in the randomness, and a more disorganized state of the adsorption system, after OME uptake by BP and BTM [31,32,46].

### 2.5. Mechanism of Adsorption

**Figure 5:**
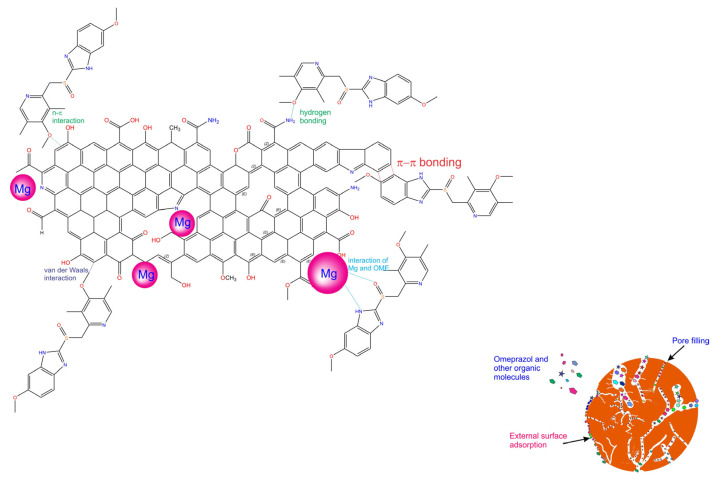
Mechanism of adsorption of OME onto BTM adsorbent.

An adsorption mechanism between OME and BTM can be stated based on the biochars’ physicochemical characteristics (porosity and chemical surface and functionalities) and adsorption results. The high adsorption of OME by the BTM adsorbent was achieved due to its physical features through the pore-filling mechanism and chemical interactions through H-bonding, π-π and n-π interactions, and the Lewis acid–base interaction [9,10,11,12,14] (see Figure 5). Since the biochars are very porous, physical adsorption through pore filling should be the principal mechanism involved in the process, although other mechanisms also take place in removing OME from the aqueous solution. In addition, the Mg on the carbon matrix generates dipoles in the carbon structure due to electronegativity differences that generate ion–dipole interaction with the OME molecules [18].

### 2.6. Synthetic Effluent Treatment Tests

**Figure 6:**
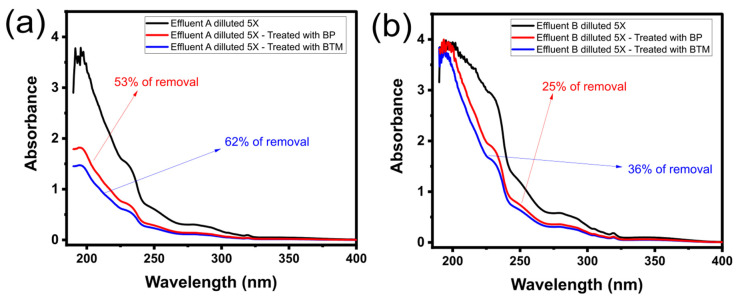
Spectra of effluents A (**a**) and B (**b**) treated by BP and BTM adsorbents.

The biobased adsorbents showed good efficiency in removing OME from aqueous solutions. Thus, they are expected to be effective in treating effluents containing several organic and inorganic compounds (similar to real effluents). Therefore, two synthetic wastewaters loaded with several drugs, and other organic and inorganic compounds (see Supplementary Table S1) were employed to test the ability of the BP and BTM to clean them up (see Figure 6).

The percentage of removal of the effluents’ compounds was measured while taking into account the UV–Vis spectra area of the two synthetic effluents before and after the treatment under the band of absorption from 190 to 500 nm [10,11,19,47] (see Figure 6). The results showed that BTM adsorbent removed 62% and 36% of the total compounds from the synthetic effluents A (lowly concentrated) and B (highly concentrated), respectively, while BP adsorbent removed 53% and 25% for effluents A and B, respectively. The better BTM efficiency in removing both effluents matches the previous adsorption results that exhibited better performances for the BTM adsorbent. These results suggest that the adsorbents prepared in this work could be employed in real effluents carried with drugs and other organic/inorganic pollutants.

## 3. Materials and Methods

### 3.1. Preparation of the Adsorbents-Activated Biochars

Spruce bark waste was used to produce the adsorbents. Firstly, the biomass waste was ground, using a hammer mill, to a particle size smaller than 0.5 mm. Then, for the doped sample (with Si and Mg), 10 g of biomass was mixed with 2 g of TEOS (tetraethyl orthosilicate) and 1 g of MgCl_2_ into a solution of water/ethanol (50%:50%), under vigorous stirring at room temperature for 2 h. The function of adding TEOS to MgCl_2_ is the possibility of forming Si-O-Mg bonds that could affect the sorption capacity of the BRM material. After that, the mixture was placed in a drying oven overnight at 80 °C. The same procedure was used for the pristine biobased material sample but without TEOS and MgCl_2_. Next, the impregnated samples were pyrolyzed using a specially designed reactor at a temperature of 800 °C in a CO_2_ flow for 1 h. After that, the temperature of the sample was increased to 10 °C/min. Finally, samples were ground using a hammer mill and sieved to a particle size < 200 μm. To wash away the remaining byproducts from the pyrolyzed carbon materials, they were washed several times with boiling de-ionized water until the pH of the wash water was equal to the pH of the de-ionized water. Hereafter, these samples were named BP (non-doped with Si/Mg) and BTM for the doped sample.

### 3.2. Characterization of the Biochars

The specific surface area (SSA) and micro/meso-pore structure were obtained using a sorption meter (Tristar 3000, Micromeritics Instrument Corp., Norcross, GA, USA). The samples were subjected to degasification at 180 °C for 3 h in a nitrogen atmosphere. The SSA was calculated using the Brunauer–Emmett–Teller (BET) method.

The adsorbents’ surface morphology was examined by scanning electron microscopy (SEM) (55-VP, Supra, Zeiss, Jena, Germany), using an acceleration voltage of 20 kV. In addition, the Raman spectra of the adsorbents were obtained using a Bruker Bravo spectrometer (Bruker, Ettlingen, Germany).

The amorphous and crystalline nature of adsorbents were evaluated through X-ray diffraction, using an X-ray diffractometer (Bruker D8 Advance, Ettlingen, Germany), operating at 45 kV and 40 mA, using Cu-Kα monochromatic radiation (λ = 1.54 Å), 2θ angle interval of 10–70 and a scan rate of 0.4°/min.

### 3.3. Adsorption Studies

De-ionized water was used to prepare the OME (omeprazole) solutions and the synthetic effluents. The batch mode was employed to evaluate the applicability of the biobased adsorbents in removing OME from aqueous solutions. In addition, the effect of the solution temperature on OME adsorption was investigated. All adsorption experiments were performed using 30 mg of each biochar into 20 mL of OME, using 50.0 mL Falcon tubes [18,19,20]. OME initial solution concentrations from 80 to 420 mg L^−1^ were employed in the experiments for isotherms studies. The kinetic OME removal on biochars was studied, varying contact time from 0 to 180 min, with an OME initial concentration of 300 mg L^−1^. The effect of temperature was measured, and thermodynamic studies were performed, at temperatures from 283 to 318 K. All adsorption experiments were performed at a constant shaking speed of 200 rpm. After adsorption, the residual solutions of OME were quantified using a UV-Visible spectrophotometer (Shimadzu 1800) at a maximum wavelength of 298 nm. The removal capacity and the percentage of OME removal are obtained from Supplementary Equations (S1) and (S2), respectively.

### 3.4. Kinetics, Equilibrium, and Thermodynamics Studies

The kinetics were studied by employing PFO, PSO, and AFO models. Three isotherm models, i.e., the Freundlich, Langmuir, and Liu models, were employed for the equilibrium study. The thermodynamics was examined from van’t Hoff approach [21], whose equilibrium constant was accessed from the best equilibrium constant obtained in the isotherms from 283 to 318 K. Information on these models and their fitness suitability are shown in the Supplementary Materials.

### 3.5. Synthetic Effluents

Two simulated pharmaceutical effluents, which consisted of drugs and other organic/inorganics compounds found in hospital wastewater, were prepared with de-ionized water [9,10,11]. The compositions and concentrations of the components of the effluents are presented in Supplementary Table S1. The purpose of using simulated effluents is to test the sorption capacities of the adsorbents for the removal of the mixture of several compounds simulating real effluents.

## 4. Conclusions

In this work, we developed adsorbents with improved emerging contaminant (OME) adsorption skills. TEOS and MgCl_2_ made carbon-based composite adsorbents as Si and Mg sources. The physicochemical characterization proved that successfully incorporating Si and Mg modified the carbon structure, yielding better adsorptive properties. The addition of Si and Mg in the carbon structure helped to increase the number of mesopores. The EDS quantification of the elements indicated 18.4% and 10.7% of Si and Mg, respectively. When tested as adsorbents, the AFO model better fitted the kinetic, and the equilibrium data were better fitted to the Liu isotherm model. The adsorbent sample doped with both chemicals (Si and Mg) presented a faster kinetic, reaching 95% of their saturations at 21.87 and 18.65 min for BP and BTM adsorbents, respectively. The maximum adsorption capacities obtained from the Liu model at 298 K were 91.54 and 249.0 mg g^−1^ for BP and BTM, respectively. The better adsorptive properties of the BTM were due to different chemical features provoked by the doping process. The thermodynamic data showed that the adsorption of OME on biobased adsorbents was spontaneous and favorable at the range of studied temperatures, with the magnitude of the adsorption corresponding to the physical adsorption process. The adsorbents were applied in treating synthetic hospital effluents containing different pharmaceuticals, organics, and inorganic salts and presented a high percentage of removal (up to 62%). The results of this work have shown that the composite between spruce bark biomass and Si/Mg was an efficient adsorbent for emerging pollutants removal. Therefore, we hope this report can help open new strategies for developing sustainable and effective adsorbents to tackle water pollution.

## Figures and Tables

**Table 1:** Textural properties of BP and BTM.

Samples	SSA (m^2^ g^−1^)	A_Mic_ (m^2^ g^−1^)	A_Mes_ (m^2^ g^−1^)	Pore Vol. (cm^3^ g^−1^)
BP	205	191	14	0.24
BTM	202	168	33	0.22

**Table 2:** OME kinetic adsorption parameters on BP and BTM adsorbents.

	BP	BTM
Pseudo-first order		
q_e_ (mg g^−1^)	71.25	75.09
k_1_ (min^−1^)	0.09760	0.1874
t_1/2_ (min)	7.105	3.699
t_0.95_ (min)	30.71	15.99
R^2^ adjusted	0.9782	0.9967
SD (mg g^−1^)	8.861	1.121
BIC	45.82	8.610
Pseudo-second order		
q_e_ (mg g^−1^)	77.86	78.82
k_2_ (g mg^−1^ min^−1^)	1.941 × 10^−3^	4.840 × 10^−3^
t_1/2_ (min)	6.613	2.622
t_0.95_ (min)	125.7	49.83
R^2^ adjusted	0.9200	0.9869
SD (mg g^−1^)	32.55	4.440
BIC	69.24	33.37
Avrami fractional order		
q_e_ (mg g^−1^)	70.23	75.42
k_AV_ (min^−1^)	0.09433	0.1977
n_AV_	1.515	0.8409
t_1/2_ (min)	8.320	3.270
t_0.95_ (min)	21.87	18.65
R^2^ adjusted	0.9969	0.9981
SD (mg g^−1^)	1.266	0.6329
BIC	12.53	0.04500

**Table 3:** Langmuir, Freundlich, and Liu isotherm parameters for omeprazole (OME) adsorption on Norway Spruce Bark adsorbents BP and BTM.

**BP**						
**Temperature in K**	**283**	**293**	**298**	**303**	**313**	**318**
**Langmuir**						
*Q*_max_ (mg g^−1^)	730.9	582.9	511.0	351.2	417	133
*K*_L_ (L mg^−1^)	68.31 × 10^−5^	7.24 × 10^−4^	5.36 × 10^−8^	1.04 × 10^−3^	7.63 × 10^−7^	1.98 × 10^−5^
*R* ^2^_adj_	0.9288	0.9278	0.8723	0.9208	0.9369	0.8992
SD (mg g^−1^)	73.30	56.63	85.57	46.20	45.32	55.98
BIC	70.40	66.53	72.72	63.47	63.18	66.35
**Freundlich**						
*K*_F_ (mg g^−1^ (mg L^−1^)^−1/nF^)	0.5770	0.4990	0.0170	0.5260	0.1780	0.1870
*n*_F_	1.054	1.061	0.659	1.119	0.901	0.9410
R^2^_adj_	0.9234	0.9220	0.9458	0.9114	0.9427	0.9012
SD (mg g^−1^)	78.79	61.22	36.29	51.69	41.16	54.85
BIC	71.48	67.70	59.85	65.16	61.74	66.05
**Liu**						
*Q*_max_ (mg g^−1^)	110.2	94.62	91.54	79.04	96.21	72.70
*K*_g_ (L mg^−1^)	0.008600	0.008400	0.005900	0.008300	0.006900	0.007270
*n*_L_	2.951	2.974	3.508	3.041	2.795	4.080
R^2^_adj_	0.9906	0.9891	0.9855	0.9852	0.9857	0.9925
SD (mg g^−1^)	9.648	8.518	9.739	8.627	10.23	4.140
BIC	41.48	39.62	41.63	39.81	42.37	28.80
**BTM**						
**Temperature in K**	**283**	**293**	**298**	**303**	**313**	**318**
**Langmuir**						
*Q*_max_ (mg g^−1^)	260.1	208.9	310.9	220.7	256.9	263.8
*K*_L_ (L mg^−1^)	3.59 × 10^−3^	4.45 × 10^−3^	1.92 × 10^−3^	4.37 × 10^−3^	4.54 × 10^−3^	3.23 × 10^−3^
R^2^_adj_	0.9839	0.9426	0.9909	0.9567	0.9784	0.9602
SD (mg g^−1^)	17.74	59.45	7.251	46.00	29.10	43.19
BIC	49.12	67.25	35.70	63.41	56.54	62.46
**Freundlich**						
*K*_F_ (mg g^−1^ (mg L^−1^)^−1/nF^)	2.603	3.207	1.314	3.1309	3.4704	2.2674
*n*_F_	1.416	1.549	1.267	1.515	1.479	1.384
R^2^_adj_	0.9721	0.9149	0.9922	0.9326	0.9649	0.9427
SD (mg g^−1^)	30.73	88.14	6.181	71.59	47.26	62.17
BIC	57.36	73.16	33.30	70.04	63.81	67.93
**Liu**						
*Q*_max_ (mg g^−1^)	146.9	107.6	249.0	112.2	152.9	118.2
*K*_g_ (L mg^−1^)	0.009500	0.01260	1.109 × 10^−8^	0.01280	0.01120	0.01140
*n*_L_	1.564	2.565	0.7892	2.404	1.566	2.276
R^2^_adj_	0.9915	0.9906	0.9916	0.9955	0.9855	0.9918
SD (mg g^−1^)	9.336	9.692	6.696	4.817	19.46	8.864
BIC	40.99	41.55	36.01	31.07	52.01	40.21

**Table 4:** Thermodynamic parameters of OME adsorption on BP and BTM adsorbents.

Temperature (K)	283	293	298	303	313	318
Liu model						
**BP**						
$K_{e}^{0}$	2980.8	2897.9	2055.1	2853.004	2379.8	2763.2
∆G° (kJ mol^−1^)	−18.82	−19.42	−18.89	−20.04	−20.23	−20.95
∆H° (kJ mol^−1^)	-	-	−1.581	-	-	-
∆S° (J K^−1^ mol^−1^)	-	-	60.90	-	-	-
R^2^	-	-	0.9930	-	-	-
R^2^_adj_	-	-	0.9895	-	-	-
**BTM**						
$K_{e}^{0}$	3274.4	4348.6	663.2	4403.9	3865.0	3947.9
∆G° (kJ mol^−1^)	−19.04	−20.41	−16.10	−21.14	−21.49	−21.89
∆H° (kJ mol^−1^)	-	-	4.022	-	-	-
∆S° (J K^−1^ mol^−1^)	-	-	81.50	-	-	-
R^2^	-	-	0.9997	-	-	-
R^2^_adj_	-	-	0.9995	-	-	-

## Data Availability

Not applicable.

## Supplementary Materials

The following are available online at https://www.mdpi.com/article/10.3390/molecules28124591/s1, “Information about Adsorption on Kinetic, Equilibrium, and Thermodynamic Models and Calculations”; Table S1: Chemical composition of simulated synthetic effluents.
