# Supplementary material for: Adsorption of Omeprazole on Biobased Adsorbents Doped with Si/Mg: Kinetic, Equilibrium, and Thermodynamic Studies
Source: Molecules. 2023 Jun 6;28(12):4591. doi: 10.3390/molecules28124591 (PMC10302240; doi:10.3390/molecules28124591)
Supplement: Supplementary file 1 [file molecules-28-04591-s001.zip › molecules-2386625-supplementary.pdf]

Supplementary material

Adsorption of omeprazole on biobased adsorbents doped with Si/Mg: Kinetic, equilibrium and thermodynamic studies.

Roberta A. Teixeira [1], Pascal S. Thue [1], Éder C. Lima [1], Alejandro Grimm [2], Mu. Naushad [3], Guilherme L. Dotto [1] and Glaydson S. dos Reis [2]*.

[1] Graduate Program in Water Resources and Environmental Sanitation, Hydraulic Research Institute (IPH), Federal University of Rio Grande do Sul (UFRGS), Porto Alegre, RS, Brazil
[2] Environmental Science Graduate Program, Engineering Center, Federal University of 8 Pelotas (UFPel), 989 Benjamin Constant St., 96010-020 Pelotas, RS, Brazil.
[3] Federal university of Rio Grand do Sul (UFRGS), Porto Alegre, RS, Brazil
[4] Department of Forest Biomaterials and Technology, Biomass Technology Centre, Swedish University of Agricultural Sciences, Umeå SE-901 83, Sweden.
[5] Department of Chemistry, College of Science, Building #5, King Saud University, Riyadh, Saudi Arabia
* Correspondence: Glaydson.simoes.dos.reis@slu.se

Kinetic of adsorption models

Pseudo-first-order: $q_t = q_e\ (1 - \exp^{-k_1 \cdot t})$ S1

pseudo-second-order: $q_t = \frac{k_2 . q_e^2 . t}{1 + q_e . k_2 . t}$ S2

Avrami fractional order: $q_t = q_{AV}[1 - exp\ (-k_{AV} t)^{nAV}]$ S3

where, ***t*** denotes the contact time (min); $\boldsymbol{q_t}$, $\boldsymbol{q_e}$ are the adsorption capacities at time *t* and at equilibrium, respectively (mg/g); $\boldsymbol{k_1}$ is the pseudo-first-order rate constant (L/min); $\boldsymbol{k_2}$ is the pseudo-second-order rate constant (g/mg min); $\boldsymbol{k_N}$ is the general-order constant rate $[(g/mg)^{n-1})/min]$, and **n** is the dimensionless general-order adsorption rate.

Equilibrium of adsorption isotherms

Langmuir: $q_e = \frac{q_{max} . K_L . C_e}{1 + K_L . C_e}$ S4

Freundlich: $q_e = K_F . C_e^{1/n_F}$

S5

Liu: $q_e = \frac{Q_{max}(K_g C_e)^{nL}}{1+(K_g C_e)^{nL}}$ S6

where, $\mathbf{q_e}$ denotes the amount of adsorbate adsorbed at the equilibrium (mg/g); C_e is the adsorbate concentration at equilibrium (mg/L); q_{max} is the maximum adsorption capacity of the adsorbent (mg/g); $\mathbf{K_L}$ and $\mathbf{K_s}$ are the Langmuir and Sips equilibrium constant (L/mg), respectively; $\mathbf{K_F}$ is the Freundlich equilibrium constant [(mg/g) $(mg/L)^{-1/n_F}$]; $\mathbf{n_F}$ and $\mathbf{n_S}$ are the dimensionless exponents of the Freundlich and Sips model, respectively.

Thermodynamic studies for the OME adsorption onto biochars were performed at temperatures ranging from 298 to 318 K.

The Gibb's free energy change (ΔG^{Θ}, kJ mol^{-1}), enthalpy change (ΔH^{Θ}, kJ mol^{-1}), and entropy change (ΔS^{Θ}, J $mol^{-1}K^{-1}$) were evaluated with the aid of Equations 7-10, respectively [13,21-26].

$$\Delta G^o = \Delta H^o - T\Delta S^o \quad \text{(S7)}$$

$$\Delta G^{\circ} = -RTLn(K_e) \quad \text{(S8)}$$

$$K_e^{\Theta} = \frac{(1000.K_g.Mw.[adsorbate]^{\Theta}}{\gamma} \quad \text{(S9)}$$

The combination of Equations 9 and 10 leads to equation 12

$$LnK_e = \frac{\Delta S^{\circ}}{R} - \frac{\Delta H^{\circ}}{R} x \frac{1}{T} \quad \text{(S10)}$$

R is the universal gas constant (8.314 J K^{-1} mol^{-1}); T is the absolute temperature (Kelvin); M_w is the molecular weight of the adsorbate (g mol^{-1}), $[adsorbate]^{\Theta}$ is the standard molar concentration of the adsorbate, which by definition is 1 mol L^{-1}; γ is the activity coefficient of the adsorbate. K_e^{Θ} is the thermodynamic equilibrium constant, calculated according to equation 16. K_e^{Θ} is dimensionless [13,21-26].

K_e^{Θ} is calculated by converting K_g values (Liu equilibrium constant) or K_L (Langmuir equilibrium constant), expressed in L mg^{-1} into L mol^{-1}. Firstly, the value K_g or K_L is multiplied by 1000 (mg g^{-1}), and then multiplied by the molecular weight of the adsorbate (g mol^{-1}) and by the standard concentration of the adsorbate (1 mol L^{-1}) and divided by the activity coefficient of the adsorbate (γ- dimensionless) [13,21-26]. It is assumed that the solution is sufficiently diluted to consider that the γ is unitary [13,21-26]. Making these calculations, K_e^{Θ} becomes dimensionless [13,21-26].

The quality control of adsorption data is further described elsewhere [13,21-26]. Nonlinear fitting of kinetic and equilibrium data was performed using the Microcal Origin 2020 software. The nonlinear fitting was obtained using the Simplex method and the Levenberg–Marquardt algorithm for performing this task. The adequacy of the kinetic and equilibrium models was statistically assessed employing the adjusted determination coefficient (R^2_{adj}), the standard deviation of residues (SD), and BIC [13,21-26] shown in equations 11, 12 and 13 below.

$$R^2_{adj} = 1 - (1 - R^2).\left(\frac{n-1}{n-p-1}\right) \quad \text{(S11)}$$

$$SD = \sqrt{\left(\frac{1}{n-p}\right).\sum_{i}^{n}\left(q_{i,exp} - q_{i,model}\right)^2} \quad \text{(S12)}$$

$$BIC = \text{nLn}\left(\frac{RSS}{n}\right) + pLn(n) \quad \text{(S13)}$$

where $q_{i,\ model}$ is the individual model sorption capacity expected by the model; $q_{i,\ exp}$ is the individual experimentally measured *sorption capacity*; $\overline{q}_{i,exp}$ is the average of all measured experimental *sorption capacities*; *n* is the number of experiments performed; *p* is the number of model parameters in the fitting model.

The R^2_{adj} and SD values were used to compare different kinetics and equilibrium models. The best-fitted model would present the R^2_{adj} closer to 1.00 and the lowest SD values [13,21-

26].

Table S1. Compositions and concentrations of efffluents containg drugs

	Concentration (mg L^{-1})	
**Pharmaceuticals**	Effluent A	Effluent B
Omeprazole	10	20
Propranolol	10	20
Amoxicillin	10	20
Captopril	10	20
Nimesulide	10	20
Diclofenac	10	20
Acetaminophen	20	40
**Sugars**		
Saccharose	30	50
Glucose	30	50
**Organic**		
Urea	10	20
Citric acid	10	20
Humic acid	10	20
**Inorganics**		
Ammonium phosphate	20	30
Ammonium chloride	20	30
Sodium sulfate	10	20
Sodium chloride	50	70
pH	6	6
